# Cause and consequences of the activated type I interferon system in SLE

**DOI:** 10.1007/s00109-016-1421-4

**Published:** 2016-04-20

**Authors:** Maija-Leena Eloranta, Lars Rönnblom

**Affiliations:** Department of Medical Sciences, Rheumatology, Science for Life Laboratory, Uppsala University, Uppsala, Sweden

**Keywords:** Type I interferon, Systemic lupus erythematosus, Plasmacytoid dendritic cells, Etiopathogenesis, Immune regulation

## Abstract

Patients with systemic lupus erythematosus (SLE) have an increased expression of type I interferon (IFN)-regulated genes (an IFN signature), which is caused by an ongoing production of type I IFNs by plasmacytoid dendritic cells (pDCs). The reasons behind the continuous IFN production in SLE are the presence of self-derived IFN inducers and a lack of negative feed-back signals that downregulate the IFN response. In addition, several cells in the immune system promote the IFN production by pDCs and gene variants in the type I IFN signaling pathway contribute to the IFN signature. The type I IFNs act as an immune adjuvant and stimulate T cells, B cells, and monocytes, which all play an important role in the loss of tolerance and persistent autoimmune reaction in SLE. Consequently, new treatments aiming to inhibit the activated type I IFN system in SLE are now being developed and investigated in clinical trials.

## Introduction

Systemic lupus erythematosus (SLE) is the prototype autoimmune disease, characterized by a very large number of different autoantibodies, immune complex formation, and organ inflammation. In addition, the majority of patients with SLE display an increased expression of type I interferon (IFN)-regulated genes, also known as an IFN signature. This observation together with previous reports that IFN-α therapy can induce an SLE syndrome suggested that the IFN signature reflects an important role of the type I IFN system in the etiopathogenesis of the disease [1, 2]. Studies during the last decade have revealed a number of environmental and genetic factors that can contribute to the ongoing activation of the type I IFN system in SLE. Furthermore, the regulation and control of the type I IFN production in SLE are disturbed with a lack of proper negative feedback mechanisms. Consequently, it has been suggested that the type I IFN system is one of the driving forces behind the disease and a number of treatment strategies aiming to downregulate IFN production in SLE have been developed. In the present review, we will mainly focus on cellular interactions involved in the activation and regulation of the type I IFN production by plasmacytoid dendritic cells (pDCs), the main type I IFN producing cell, and how an ongoing activation of the type I IFN system can contribute to the SLE disease process.

## Systemic lupus erythematosus

Systemic lupus erythematosus (SLE) is one of the most heterogeneous autoimmune diseases with multisystemic presentation and a wide range of clinical and serological manifestations [3, 4]. The disease varies between individual patients from relatively mild manifestations of skin and joints to life-threatening renal and central nervous system involvement [4]. Besides clinical heterogeneity, SLE patients also demonstrate immunological heterogeneity, indicating multiple pathogenic mechanisms. Consequently, patients with SLE can respond very differently, or not at all, to the same therapeutic regimen [5].

More than 200 different autoantibodies have been described in SLE, which partly may explain the diverse clinical phenotypes [6]. However, the most commonly found autoantibodies are directed against single-stranded (ss) and double-stranded (ds) DNA, Ro/La antigens, and ribonuclear protein (RNP), which can be present years before the clinical onset of SLE [7]. Some of the autoantibody specificities have been associated with distinct clinical manifestations or disease activity, e.g., anti-dsDNA and anti-Ro antibodies correlate with renal disease and photosensitive skin rash, respectively [8].

Patients with SLE have an ongoing IFN-α production, which gives rise to the IFN signature that can be demonstrated in approximately 50 % of adult SLE patients and the majority of children with SLE [9]. Several studies have found an association between the IFN signature and multiple clinical manifestations, such as nephritis and CNS engagement [10, 11], and recent data suggest that patients with a high type I IFN signature represent a distinct subset of SLE patients that respond to type I IFN blockade, see below.

## Activation of type I interferon production

The type I IFN family comprises 13 IFN-alpha (-α) subtypes and one copy of IFN-β, IFN-ε, IFN-κ and IFN-ω, respectively. Normally, the IFN-α and IFN-β production is strictly controlled but starts rapidly when viral or bacterial nucleic acids are sensed by pattern recognition receptors (PRRs), such as certain Toll-like receptors (TLRs) or cytoplasmic nucleic acid sensors [12]. The route activated depends on the PRR repertoire of the responding cell type and the subcellular localization of the immunostimulatory nucleic acid.

The TLRs involved in the type I IFN production are mainly present in immune cells where they are located in the endolysosomal compartment sensing double-stranded (ds) RNA (TLR3), single-stranded (ss) RNA (TLR7/8), and dsDNA containing unmethylated CpG motifs (TLR9) [13]. The latter is also reported to sense RNA/DNA hybrids which are generated during replication of several types of viruses [14].

Two cell types that are capable of secreting large amounts of IFN-α and IFN-β are plasmacytoid dendritic cells (pDCs) and monocytes. The latter respond mainly to dsRNA and certain RNA viruses such as Sendai and influenza virus, whereas pDCs can be triggered to secrete type I IFN by almost all viruses and some bacteria [15]. pDCs express TLR3, TLR7, and TLR9 and have a high basal level of interferon regulatory factor (IRF) 7, which contribute to the rapid and massive onset of IFN-α production (1–3 pg per cell) [16]. Another group of molecules important for initiating the type I IFN production consists of cytoplasmic RNA sensors such as RNA helicases that are expressed by many different cell types. In addition, several cytosolic DNA sensors have recently been described including DNA-dependent activator of IFN-regulatory factor 1 (DAI) and cGMP-AMP synthase (cGAS), with suggested roles in recognizing pathogen-derived dsDNA as well as incompletely digested self-DNA [17, 18]. Taken together, there seems to be a redundancy of nucleic acid sensing immune receptors, and it remains to be clarified how these are involved in the recognition of DNA and RNA of endogenous origin and what the consequences are if they are not regulated properly.

## Type I interferon production in SLE

Several possible mechanisms behind the ongoing type I IFN production in SLE have been implicated during the last years. One important mechanism of IFN-α induction in SLE is mediated by interferogenic ICs, which are immune complexes consisting of autoantibodies and nucleic acid-binding proteins that are endocytosed through FcγRIIa on pDCs and transported into the endosomes where the nucleic acid part of the IC interacts with TLR7 or TLR9 [19, 20]. Type I IFN production by pDCs can also be trigged by neutrophil extracellular traps (NETs) [21]. The NETosis is a relatively recently described cell death pathway where neutrophils extrude nuclear material such as decondensated chromatin, histones, granular, and cytoplasmic proteins in a web-like structure which can entrap invading pathogens. It was shown that a subset of SLE patients have an impaired capacity to degrade NETs due to decreased function of extracellular DNAse I, which increases the exposure of nucleic acids and proteins available to autoantibodies and autoreactive B cells [22, 23]. This could also lead to further stimulation of pDCs to produce IFN-α. Recently, Lood et al. demonstrated that mitochondria-derived ROS initiated NETosis and release of oxidized mtDNA which can induce IFN-α via activation of the cytosolic cGAS stimulator of interferon genes (STING) pathway or via the endosomal TLR9 pathway [24]. In summary, multiple immune mechanisms have evolved to react to nucleic acids in different cellular compartments, and one of the critical risk factors for SLE could be the increased exposure of nuclear contents to such nucleic acid sensors.

## Signaling via the type I IFN receptor

All type I IFNs bind, albeit with slightly different affinity, to the same heterodimeric type I IFN receptor (IFNAR1) expressed on the cell surface of most cell types [25]. Binding of type I IFNs to IFNAR1 initiates signaling cascades through multiple pathways. The most thoroughly characterized type I IFN signaling pathway is the Janus activating kinase (JAK)-signal transducer and activator of transcription (STAT) pathway involving phosphorylation of cytoplasmic JAK1 and tyrosine kinase (TYK) 2, and subsequently STAT 1 and 2. A complex of activated STAT1 and 2 together with IRF9 translocates to the nucleus where it binds to IFN regulatory elements and triggers transcription of several hundreds of type I IFN-stimulated genes (ISGs) [25, 26]. Other members of the STAT family, e.g., STAT3, 4, and 5 can also be activated by type I IFNs but instead bind to IFN-γ-activated response (GAS) elements. In addition, other signaling pathways such as MAPK (p38 and ERK) and PI3K/AKT can be activated by IFNAR engagement and either cooperate with the JAK/STAT pathway or act independently to trigger expression of ISGs [27].

## Environmental factors triggering IFN production in SLE

There are a number of environmental factors that can both induce an SLE syndrome and trigger a flare of the disease. Several of these factors are by various mechanisms potent activators of the type I IFN system. Ultraviolet (UV)-B light is perhaps the most well-known environmental trigger of SLE flares and can induce severe systemic manifestations beyond the cutaneous reactions in photosensitive patients [28, 29]. Multiple mechanisms are involved in the UV light-induced exacerbation of SLE, but important are the induction of type I and III IFNs as well as chemokines. Type III IFNs acts via a separate receptor but induce similar effects as type I IFNs and are important for the protection of epithelial cells against viruses [30]. Thus, UV light (290–320 nm) exposure causes redistribution of nuclear antigens to be exposed on the cell surface and also triggers apoptosis and secondary necrosis of keratinocytes [29]. In this way, normally hidden autoantigens, such as nucleoproteins, can be recognized by autoantibodies and form interferogenic immune complexes that induce type I IFN production by pDCs in the skin [29]. In addition, UV light can induce release of reactive oxygen species which cause DNA strand breaks and pyrimidine dimer formation in DNA further facilitating the availability of the nucleic acids for IC formation [28].

In SLE, the pDCs are localized in peripheral organs, such as skin and kidneys, where they are exposed to interferogenic ICs, chemokines, and other stimulatory cytokines [16]. For example, it has been shown that keratinocytes from patients with cutaneous lupus and several other inflammatory skin diseases produce high levels of type III IFNs [31]. The keratinocyte-derived type III IFNs and the chemokine CXCL9 recruit more inflammatory cells into the skin but probably also “prime” the activated pDCs to enhanced type I IFN production. Recently, it was discovered that UV irradiation of keratinocytes enhanced the activation of the STING/IRF3 signaling pathway in response to cytosolic DNA due to loss of Unc51-like kinase 1 which is a negative regulator of STING [32]. Such mechanism could lead to upregulated type III IFN production and priming of pDC function in the presence of RNA recognized by TLR3. Taken together, sun exposure can trigger and enhance the type I IFN production in the skin of SLE patients leading to local as well as systemic effects and increased disease activity.

A large number of drugs have been reported to induce an SLE-like syndrome (drug-induced lupus: DIL) with various clinical and serological symptoms that dissolve when the medication is withdrawn [33]. The most well-known drugs inducing DIL are procainamide and hydralazine, reviewed in [33]. Procainamide is an inhibitor of methyltransferase and prevents DNA methylation, which can affect the regulation of gene expression. Interestingly, recent studies have revealed that methylation of nonhistone proteins is a highly dynamic process which together with phosphorylation regulates signal transduction as shown for example for STAT3 in the JAK/STAT pathway [34]. In addition, antibodies to chromatin and nucleosomes are commonly found in DIL as well as reduced clearance of apoptotic cells [33].

It is common that infections trigger the onset of SLE or a disease flare. Even though many viruses have been implicated in the SLE etiology, no specific virus has been identified to cause the disease. As microbial RNA and DNA can be recognized by multiple nucleic acid sensors and thereby induce production of type I IFNs, this may be the mechanism whereby several microorganisms can contribute to the development and relapse of SLE.

## The genetic background to SLE

SLE has a strong familial aggregation and a higher disease concordance rate between monozygotic twins (24–40 %) compared to dizygotic twins or other siblings (2–5 %) [35, 36]. Genome-wide association studies (GWAS) have identified more than 50 SLE-associated genetic loci, most of which affect pathways implicated earlier in SLE etiopathology, such as immune complex processing, Toll-like receptor signaling, and type I IFN production/response [37–39]. Notably, more than half of the loci are connected to the type I IFN system [40], including *IRF5*, *TYK2*, and *STAT4*, which are central for activation of the type I IFN production and signaling [27]. However, the exact function of most of the risk gene variants is unknown, but some gene variants were shown to correlate with a certain phenotype, such as the risk *IRF5* haplotype that is associated with increased serum IFN activity in SLE patients [41]. Surprisingly, we found that the *IRF5* risk haplotype was associated with a lower IFN-α production in pDCs from healthy individuals stimulated with RNA-IC, compared to the production by pDCs with the protective haplotype [42]. This could be interpreted as a result of the disease-specific microenvironment in SLE patients compared to healthy individuals. The conclusion to be drawn from the study is that SLE risk variants can either contribute to increased or decreased type I IFN production, but the net effect is determined by the combined effect of a large number of gene variants.

Although SLE is considered as a complex disorder, rare SLE cases with a Mendelian mode of inheritance have been described [40, 43]. Some of these monogenic SLE diseases are now categorized as type I interferonopathies, due to the prominent type I IFN signature.

The most well-known monogenic defects associated with a high risk for SLE are loss-of-function mutations in *C1q* and *C4,* encoding components of the classical complement pathway, and in the 3′-5′ exonuclease *TREX1* [44, 45], the latter leading to accumulation of intracellular DNA that triggers type I IFN production. The complement system is important in the clearance of immune complexes, and it has been shown that C1q inhibits the production of IFN-α and several other cytokines by pDCs [46, 47], which could explain the increased type I IFN production in C1q deficiencies. Although SLE-associated risk alleles of C1q, C4, and TREX1 are rare in the population, they confer a high relative risk for SLE.

## Effects of type I IFN on the immune system

Type I IFNs have a broad spectrum of effects on innate and adaptive immune responses [10, 48], but the actual mode of action is dependent on the responding cell type as well as the cellular and genetic context [49]. Also, the effects of IFN subsets vary, probably due to a differential binding to the IFNAR receptors subunits [50].

In addition to the direct antiviral effects, both IFN-α and IFN-β efficiently enhance the effector capacity of natural killer (NK) cells and macrophages against intracellular microbes in the first-line immune defense [51]. In addition, expression of MHC I molecules is increased by type I IFN on several cell types, which facilities the cross-presentation of exogenous antigens as well as detection of virus infected cells by cytotoxic T cells [52]. See Table 1.

**Table 1:** Effects of interferon-alpha

Target cell	Effects
NK cells	Increased cytolytic activity [51]
Macrophages	Enhanced intracellular killing of pathogens and expression of co-stimulatory molecules [51]
Dendritic cells	Maturation, enhanced antigen presentation [49]
Plasmacytoid DC	Enhanced type I IFN production, homing to lymph nodes [15, 16, 53]
CD4+ T cells	Prolonged survival, promotion of Th1 profile, increased IL-12R expression, generation of memory cells [49]
CD8+ cytotoxic T cells	Enhanced cytotoxity, inhibition of apoptosis [49]
Regulatory T cells	Suppression of Treg activity [49, 54, 55]
Th17 T cells	Skewing of Th cells towards Th17 profile and IL-17 production [49, 54, 55]
B cells	Increased plasma cell differentiation, isotype switch, and enhanced antibody production, generation of memory cells [56, 57]
Endothelial cells	Induction of apoptosis, impaired regeneration [58, 59]

IFN-α promotes the expression of MHC II and co-stimulatory molecules, such as CD40, CD80, CD86, and production of several cytokines stimulating the differentiation of monocytes and immature DC into effective antigen presenting cells [51]. An increased expression of chemokines and their cognate receptors such as CXCL10 and CXCR3 direct cells to the sites of inflammation, which is demonstrated by a reduced number of pDCs in the peripheral blood of SLE patients [60].

With regard the adaptive immunity, type I IFNs prolong the survival of activated T lymphocytes and stimulate the development of CD4+ and CD8+ memory T cells. In addition, type I IFN increase the differentiation of Th17 cells and suppress Treg functions, which all can lead to an expansion of autoreactive T cells and enhanced inflammatory responses [54].

Concerning the effects on B cells, type I IFNs increase the production of B-lymphocyte stimulator (BLyS), B cell proliferation and lower the threshold required for activation through the B cell receptor. The antibody production is effectuated via increased immunoglobulin isotype class switch, differentiation into plasma cell, and enhanced antibody production [55–57].

The type I IFNs have also effects outside the immune system, and an important one in the SLE context is the impairment of endothelial function by induction of apoptosis and slowing down the repair process of damaged endothelium [58]. One can speculate that pDCs activated by interferogenic ICs and inflammatory NETs formed in situ within the blood vessel could be linked to the unexpectedly high prevalence of atherosclerosis and cardiovascular disease in young female SLE patients [59].

Clearly, a persistent synthesis of type IFNs has many crucial effects on the immune system that can contribute to the loss of tolerance and exacerbate the immune pathology in individuals prone to autoimmune disorders.

## Regulation of the IFN system in SLE

**Fig. 1:**
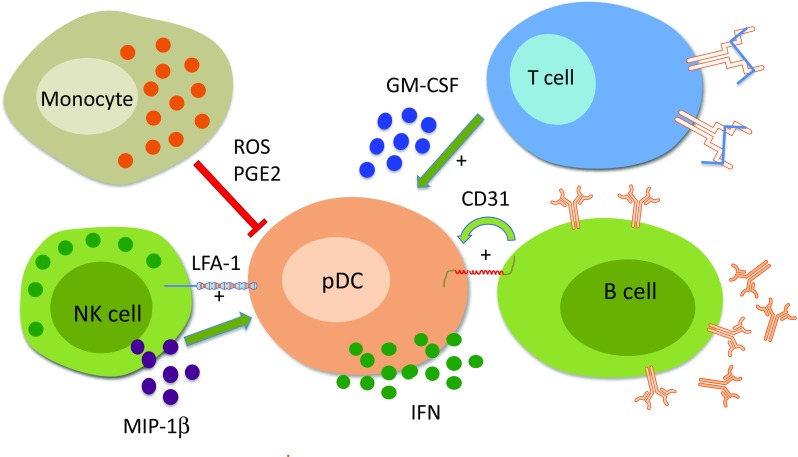
Type I interferon production is affected by interactions between plasmacytoid dendritic cells and other cell types. Plasmacytoid dendritic cells (pDCs) produce type I interferon (IFN) when stimulated with RNA containing immune complexes (RNA-IC). Activated monocytes/macrophages suppress the capacity of pDCs to produce type I IFN by releasing reactive oxygen species (ROS) and prostaglandin E2 (PGE2). In contrast, NK cells enhance the type I IFN production by activated pDCs via lymphocyte-associated antigen (LFA)-1 and secretion of MIP-1β. Also, the B cells and activated T cells increase the type I IFN production by RNA-IC-stimulated pDCs via a mechanisms involving CD31 molecule and soluble GM-CSF, respectively

Normally, the IFN-α production is terminated after the pathogen has been eradicated and pDCs become temporally refractory to new stimuli due to inhibition and degradation of transcription factors and signal transducers [53, 61]. The stability of type I IFN mRNAs themselves can also be regulated by micro-RNAs or factors binding to AU-rich elements [61]. However, the impact of cellular communication on the regulation of the type I IFN production is largely overlooked. We have therefore investigated several aspects of the cellular cross-talk and showed that interactions between pDCs and monocytes, NK cells, B cells, and activated T cells in a complex network can modulate the type I IFN production by pDCs (see Fig. 1). We previously demonstrated that monocytes from healthy individuals effectively reduced the IFN-α production by pDCs stimulated with RNA-containing ICs consisting of U1 snRNP and SLE-IgG (RNA-IC). The suppression was mediated via reactive oxygen species, TNF-α, and prostaglandin E2 [62]. Interestingly, monocytes isolated from patients with SLE did not have such strong suppressive capacity and could represent a component contributing to the dysregulated type I IFN system in the SLE.

In contrast to monocytes, it was shown that NK cells, B cells, and activated T cells have the capacity to strongly enhance the RNA-IC-stimulated IFN-α production by pDCs [62–64]. The mechanism for the stimulatory effect of NK cells involves the production of adhesion molecule LFA-1 and MIP-1β the latter triggered via FcRγIIIa (CD16) on NK cells [65]. The stimulatory effect was entirely mediated by CD56^dim^ CD16+ NK cells, but the CD56^bright^ CD16+ NK cells became equally effective after exposure to IL-12/IL-18. In comparison, NK cells isolated from patients with SLE had a reduced stimulatory effect on the IFN-α production by pDCs, but in similar manner as the CD56^bright^ CD16+ NK cells from healthy individuals, the patient NK cells became stimulatory when cultivated with IL-12 and IL-18 [65]. The stimulatory capacity of the B cells on the IFN-α production by RNA-IC activated pDCs was dependent on direct cell contact and was abolished by blocking the endothelial cell adhesion molecule (PECAM-1/CD31) [63]. The CD31 is a cell surface receptor expressed on both B cells and pDCs [63, 66] and the cytosolic part of the CD31 contains two tyrosine-based inhibitory motifs (ITIMs). Interestingly, it was recently reported that CD31 acts as a co-inhibitory receptor in activated conventional DCs and prevented formation of functional immunological synapses between T and B cells [67]. In contrast, when a TLR9 analog was used as stimulus there was no requirement for direct cell contact or involvement of CD31 for the enhancing effect of B cells. The difference of CD31 involvement could have implications in situations when the goal is to block the type I IFN production triggered by RNA containing IC via TLR7 while leaving the TLR9 pathway intact. We recently demonstrated that also activated T cells have the capacity to enhance the IFN-α production by RNA-IC stimulated pDCs [64]. After pre-activation, T cells from SLE patients and from healthy individuals were equally effective in their stimulatory capacity [64]. The enhancing effect on the IFN-α production by pDCs was largely mediated by the cytokine GM-CSF and to a lesser extent by IL-3 [64]. This could reflect the in vivo situation in a subset of SLE patients with the presence of hyperactivated T cells and increased levels of GM-CSF [64].

In summary, the type I IFN production by pDCs and other cell types can be enhanced or suppressed by several different mechanisms. Obviously, several components in the cellular pathways involved in type I IFN signaling, recognition of nucleic acids, and clearance can either increase or decrease the levels of type I IFN. How the pDCs escape the negative feedback mechanisms in SLE and other disorders with persistently activated type I IFN system remains to be further clarified.

## Therapeutic options

There are several possibilities to downregulate the type I IFN system in SLE, and at the moment, a number of clinical trials are in progress, reviewed in [5]. Among standard therapies for SLE, both high doses of glucocorticosteroids and hydroxychloroquine downregulate the IFN signature, but today, more specific inhibitors of the type I IFN system exist. Thus, three different monoclonal anti-IFN-α antibodies have been developed, which at least partially decrease the IFN signature and disease activity [5]. A more complete inhibition of the type I IFNs is to target the IFNAR, and recently, the first results from treatment of SLE with an anti-IFNAR antibody were reported [68]. In this study, there was an ∼90 % suppression of the IFN signature and a significant improvement in disease activity, but at the cost of an increased frequency of viral infections. New therapeutic strategies are to degrade the stimulatory TLR ligands in interferogenic ICs by nucleases or to use inhibitory oligodeoxynucleotides to block TLR activation [69]. A broader therapeutic approach is to target the pDCs themselves with monoclonal antibodies against BDCA-2 [70], or to use proteasome inhibitors which efficiently suppress production of IFN-α by TLR-activated PDCs [71]. When the exact mechanisms behind the IFN signature in individual patients can be identified, more specific drugs for the relevant pathogenic pathway can be expected to be developed. One possibility could be administration of reverse transcriptase inhibitors for SLE patients with chronic stimulation of DNA sensors by retroelement cDNA [72]. A more personalized approach to modulate the type I IFN system in order to reduce the risk for increased frequency and severity of infectious diseases would be a major therapeutic leap forward for this vulnerable group of patients.
